# A New Model of Alcoholic Fermentation under a Byproduct Inhibitory Effect

**DOI:** 10.1021/acsomega.0c04025

**Published:** 2021-02-01

**Authors:** Hamid Zentou, Zurina Zainal Abidin, Robiah Yunus, Dayang R. Awang Biak, Mohammed Abdullah Issa, Musa Yahaya Pudza

**Affiliations:** Department of Chemical and Environmental Engineering, Universiti Putra Malaysia, Serdang 43400, Malaysia

## Abstract

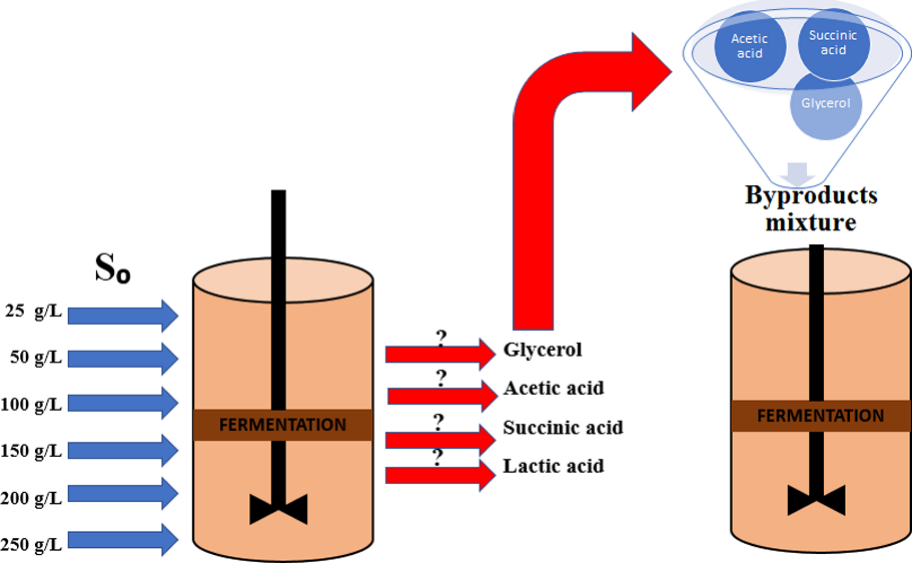

Despite the advantages of continuous fermentation whereby ethanol is selectively removed from the fermenting broth to reduce the end-product inhibition, this process can concentrate minor secondary products to the point where they become toxic to the yeast. This study aims to develop a new mathematical model do describe the inhibitory effect of byproducts on alcoholic fermentation including glycerol, lactic acid, acetic acid, and succinic acid, which were reported as major byproducts during batch alcoholic fermentation. The accumulation of these byproducts during the different stages of batch fermentation has been quantified. The yields of total byproducts, glycerol, acetic acid, and succinic acid per gram of glucose were 0.0442, 0.023, 0.0155, and 0.0054, respectively. It was found that the concentration of these byproducts linearly increases with the increase in glucose concentration in the range of 25–250 g/L. The results have also showed that byproduct concentration has a significant inhibitory effect on specific growth coefficient (μ) whereas no effect was observed on the half-velocity constant (*K*_s_). A new mathematical model of alcoholic fermentation was developed considering the byproduct inhibitory effect, which showed a good performance and more accuracy compared to the classical Monod model.

## Introduction

**Figure 1:**
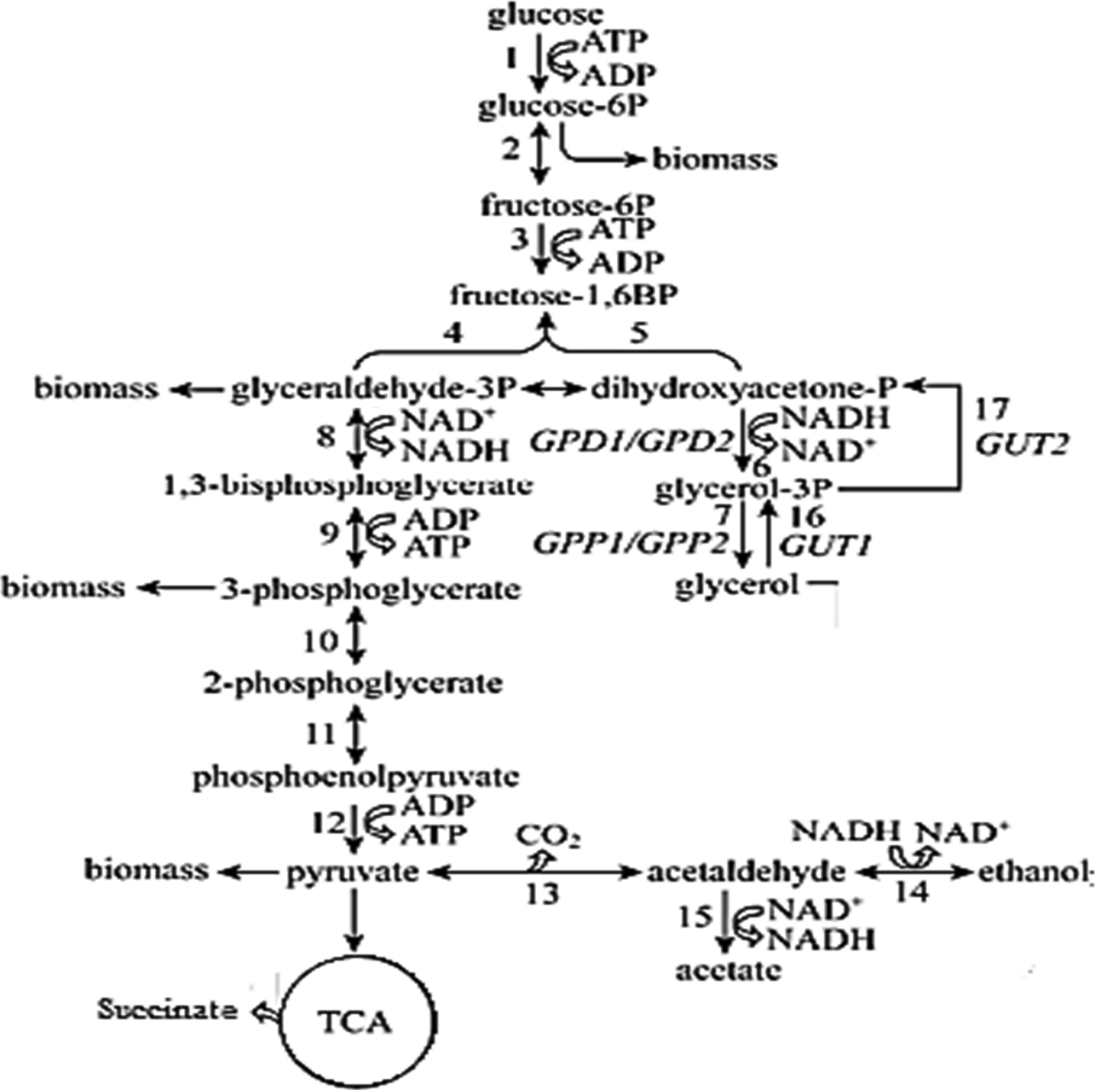
Byproduct formation pathways during alcoholic fermentation. Adapted with permission from ref (14). Copyright 2011 Springer.

Increasing concern about energy security and environmental issues such as emission of greenhouse gases has raised interest in the development of renewable bioenergy as an alternative energy to fossil-based fuel.^1−3^ There are two main industrial sectors in biofuel production, namely, bioethanol and biodiesel. Bioethanol can be produced by the fermentation of sugars, whereas biodiesel is derived from vegetable or animal fat through the process of transesterification.^4^ In this regard, the fermentation of agricultural residues and industrial wastes for bioethanol production becomes a promising alternative for the production of ecofriendly energy with a low cost.^5−7^ Therefore, growing attention has been devoted to the optimization of the fermentation process to increase the yield and to minimize the production cost, which, in turn, will promote the bioethanol industry and help overcome the associated challenges.^8^ Batch fermentation systems are preferred for industrial applications as they limit the risk of contamination and do not need high capital investment as they do not require expensive production equipment compared to continuous processes. However, the batch fermentation process can be unaffordable particularly in the downstream ethanol recovery process, since if the product titer is low (<4%), the cost of distillation is too high.^9^ Continuous fermentation whereby ethanol is selectively removed from the fermentation broth seems an ideal choice with a high ethanol productivity and limited inhibition of the end product and substrate. However, this process can concentrate minor secondary products to the point where they become toxic to the yeasts.^10,11^ Many growth and environmental factors have been reported to influence the nature of byproducts produced during alcoholic fermentation such as sulfite concentration, pH, fermentation temperature, aeration, and inoculation level.^12^ Glycerol and organic acids (acetic acid, lactic acid, tartaric acid, and formic acid) and higher alcohols are quantitatively the most important fermentation products after ethanol and carbon dioxide.^11^ Short-chain weak organic acids are potent inhibitors of microbial growth during industrial fermentation processes as the accumulation of these fermentation byproducts may suppress the ultimate productivity of ethanol and microbial growth.^13^ The formation of these byproducts is driven by different factors such as microorganism (yeast) response when adapting with the exterior environment via different pathways, as illustrated in Figure 1.

Due to the importance of mathematical modeling as a tool that helps in process control, reduction in production costs, and improvement in the product quality of the fermentation, different models have been developed to include product inhibition,^15−19^ substrate inhibition,^15,19−21^ and the inhibition of cell density called “self-inhibition“.^22,23^ On the other hand, despite the significant inhibitory effect of byproducts especially glycerol and organic acids, which has been reported in several investigations,^11−13,24,25^ there are scarce studies about the mathematical models that consider the inhibitory effect of byproducts. Thus, a vital part of the present study was devoted to develop a new model based on the Monod model while at the same time take into account the inhibitory effect of byproducts. In addition to that, the effect of substrate concentration on byproduct formation during alcoholic fermentation will also be discussed.

## Experimental Section

### Yeast Strains and Media Preparation

Three loops of active dry *Saccharomyces cerevisiae* yeast from Saf-Levure (Lesaffre, Marcq, France) were dissolved in 50 mL of distilled water, which were then added directly into 200 mL of yeast peptone dextrose (YPD) culture media containing 20 g/L glucose, 20 g/L peptone, and 10 g/L yeast extract supplied by Sigma-Aldrich (M) Sdn Bhd, Malaysia.^26^ The fermentation medium was incubated at 35 °C and shaken at 250 rpm for 6 h under aerobic conditions. The used chemicals and materials were sterilized in an autoclave at 121 °C for 20 min before the experiment.

### Fermentation Process and Experimental Design

To elaborate the relationship between the substrate consumption and byproduct production to eventually determine the mathematical equation representing the accumulation of the major byproducts with time and substrate consumption, a duplicate fermentation run was carried out. The following metabolites were considered as major byproducts during fermentation of glucose based on the works of Maiorella et al.: glycerol, lactic acid, acetic acid, and succinic acid.^11^

**Figure 2:**
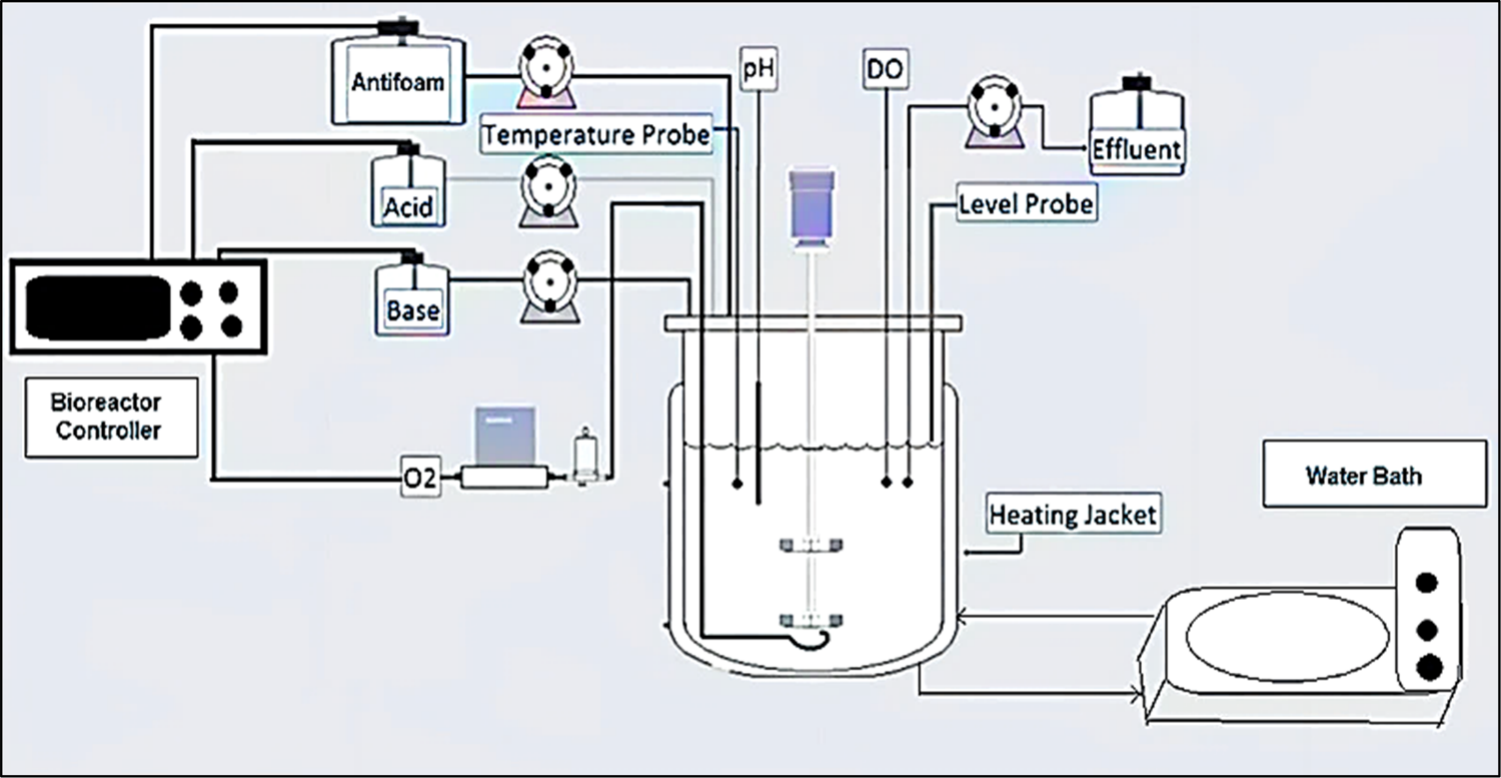
Schematic of the bioreactor for a batch fermentation operation.

The fermentation was carried out in a 2 L stirred tank fermenter (BIOF-2 L model, Labfreez), with a working volume of 1 L (Figure 2). To study the effect of glucose concentration on byproduct formation, fermentation media (1 L) with different initial glucose concentrations (25–250 g) have been prepared. Yeast was added to the prepared fermentation medium with a concentration of 1 g/L (calculated as fresh baker’s yeast). During the fermentation process, the pH value was adjusted at pH = 4.5 by the automatic addition of 0.1 M NaOH and the stirring speed was maintained at 250 rpm. The fermentation temperature was kept at 30 °C using a water jacket.^27^ The fermentation was carried out under microaeration conditions (1 vvm) for 2 h and turned later to anaerobic during the rest time of fermentation. A sample of 5 mL was taken at a predetermined time (0, 2, 4, 6, 8, 10, 12, 18, 24, 30, 36, 48, and 72 h) in order to determine the concentration of sugars, ethanol, biomass, and byproduct content (glycerol, lactic acid, acetic acid, and succinic acid).

To investigate the byproduct effect on the alcoholic fermentation process, a mixture model of the previous byproducts with different concentrations was added to the initial medium (25 g/L glucose, 20 g/L peptone, and 10 g/L yeast extract). The composition of the mixture and each byproduct fraction in the mixture was determined based on the results of the first experiment.

### Analytical Methods

The yeast concentration was determined using a spectrophotometer at 620 nm and a calibration curve of cell dry weight measurements versus the absorbance. The concentrations of organic acids, glycerol, glucose, and ethanol were evaluated with HPLC using a Bio-Rad Aminex HPX-87H column, as described in NREL laboratory methods.^28^ A volume of 1–2 mL of the sample was supplemented with redistilled water and then filtered with a 0.45 μm PTFE syringe filter. The analysis was performed with an HPLC system designed by Shimadzu, which is equipped with a Bio-Rad Aminex HPX-87H (300 mm × 7.8 mm) column and intelligent refractive index for detection. The mobile phase was 0.005 N H_2_SO_4_ with a flow rate of 0.5 mL/min and the temperature was kept at 65 °C.

### Mathematical Theory and Modeling

The main objective of the present work is to develop a new model considering the byproduct inhibitory effect during alcoholic fermentation. The suggested model will be based on the modification of the Monod model where the following four steps have been conducted.

1. Chemical analysis to determine the major byproducts in the fermentation broth and to prepare the standard model of the byproduct mixture.
2. Studying the effect of substrate concentration (glucose) on byproduct formation to determine the byproduct yield coefficient called (*Y*_*z*/*s*_).
3. Determination of the byproduct concentration effect on the fermentation process in terms of specific growth coefficient μ_max_ and the half-velocity constant *K*_s_ and identification of *Z*_max_, the maximum byproduct concentration that stops the fermentation process totally.
4. Modeling of the byproduct inhibitory effect and simulation of the new model to be compared to the experimental data.

The Monod equation describes the dependence of microorganism’s growth rate on the concentration of a limiting substrate:^29^
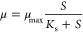
1where μ_max_ is the maximum specific growth rate (h^–1^), *S* is the concentration of the growth limiting substrate (g/L), and *K*_s_ is the half-velocity constant, i.e., the substrate concentration.

Considering the byproduct inhibitory effect, the specific growth rate under inhibition can be written as follows:
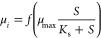
2

*f*(*x*) is the function of the variance of μ versus the byproduct concentration (*z*).
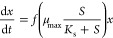
3
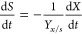
4
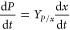
5
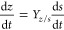
6where *Y*_*x*/*s*_, *Y*_*p*/*s*_, and *Y*_*z*/*s*_ are the biomass, product, and byproduct specific yield coefficients.

Thus, the new modified model can be developed once the inhibition function *f*(x) is identified and the yield coefficients *Y*_*x*/*s*_, *Y*_*P*/*x*_, and *Y*_*z*/*s*_ are calculated.

### Model Simulation and Validation

The models were solved and simulated using the fourth-order Runge–Kutta method ODE 45 with the MATLAB R2014a software. The models’ performance was statistically assessed with the coefficient of determination (*R*^2^) using the OriginPro 8.5 software where the simulation data were validated and compared to the experimental data set that was not used for parameter estimation.

s

## Results and Discussion

### Monitoring of Byproduct Formation during Alcoholic Fermentation

**Figure 3:**
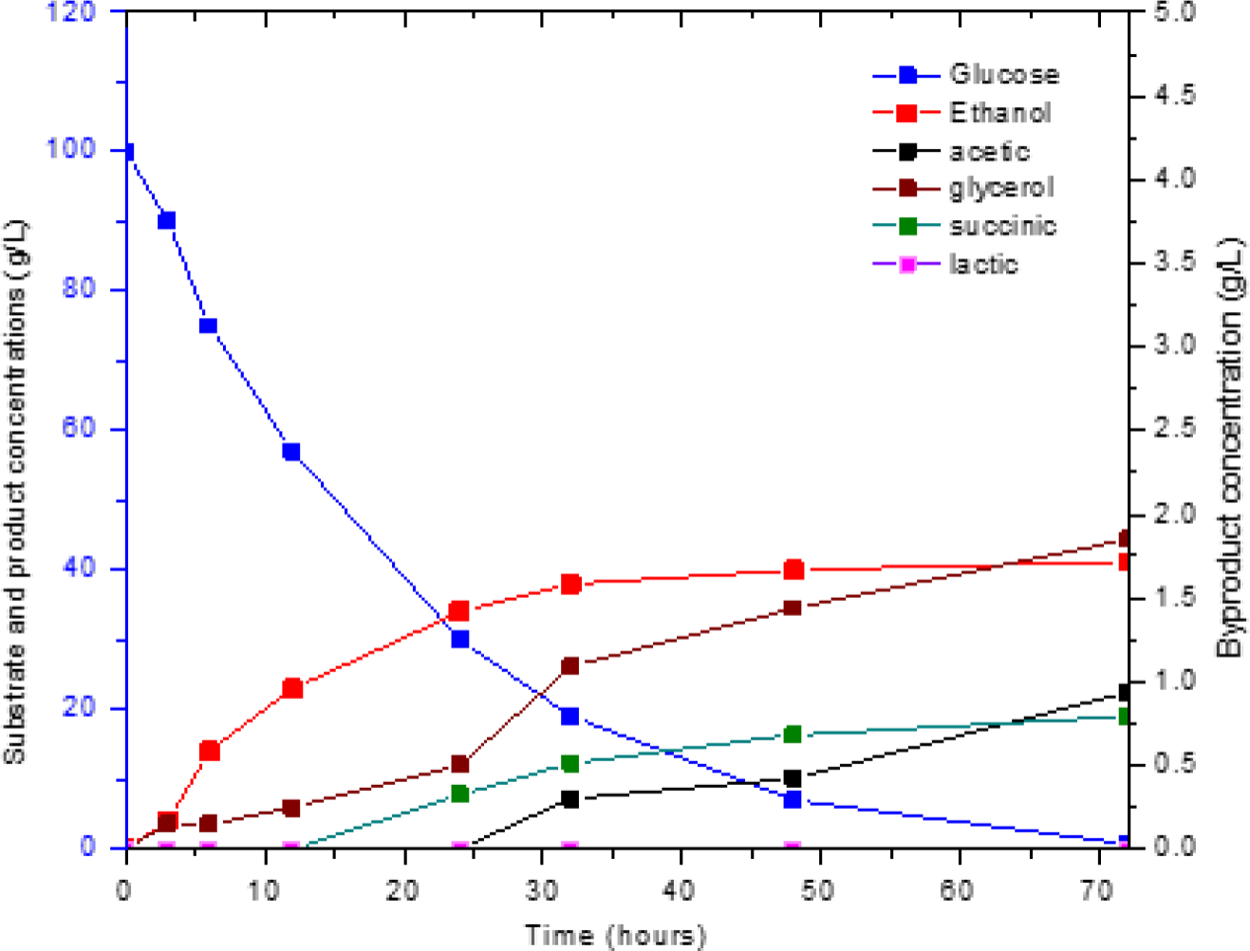
Byproduct formation during the batch fermentation process (*S*_0_ = 100 g/L) as a function of time.

To investigate the formation of byproducts during different stages of fermentation, batch fermentation was carried using 100 g/L glucose solution for 72 h. Samples were taken within a predetermined time to measure the concentration of glucose, ethanol, and different byproduct concentrations including glycerol, acetic acid, succinic acid, and lactic acid. These selected byproducts are compounds that are believed to reach their inhibitory levels quickest in comparison to other byproducts based on a previous study.^11^Figure 3 represents the monitoring of the byproduct formation during alcoholic fermentation for an initial glucose concentration *S*_0_ = 100 g/L. The results showed the absence of lactic acid in all tested samples, which support the concept that lactic acid is a byproduct of abnormal alcoholic fermentation and may be considered an indicator of bacterial contamination in the broth.^30−33^ Glycerol was the major byproduct, which represented more than 50% of the total mass of byproducts in all samples. The formation of glycerol was noted in the first stage of fermentation until the end where the final concentration of glycerol was 1.8 g/L. However, the formation of glycerol was more accelerated in the first stage than in the final stage of fermentation. This is expected due to the high concentration of glucose in the first stage of the fermentation, which increases the osmotic stress during the first stage of fermentation; in turn, the yeast produces more glycerol as the main osmo-protectant to minimize the effect the osmotic stress.^34^ Glycerol is mainly formed in two steps: reduction of dihydroxyacetone phosphate to form glycerol-3-phosphate, which is then dephosphorylated to produce glycerol.^35^ It is also found that the formation of acetic acid was associated with the glycerol accumulation. A concentration of 1 g/L glycerol represents a point of departure of acetic acid formation, which continues until the end of fermentation with 0.95 g/L concentration. In addition to being a substrate for acetyl-CoA synthetase, a physiological role of acetate formation may be the regeneration of reducing equivalents (NADH and NADPH) for maintaining the redox balance.^12^ Succinic acid appeared in the broth after 24 h of fermentation and progressively increased until the end of fermentation, achieving a concentration of 0.8 g/L. The total tested byproduct mixture concentration was around 3.55 g/L, which is in agreement with the results reported in previous studies where the weight fractions of glycerol, acetic acid, and succinic acid in the mixture were 56, 36, and 8%, respectively.^11,36^

### Effect of Glucose Concentration on Byproduct Formation

All the byproducts presented in this study showed a dependency on glucose concentration especially glycerol. The increase in glycerol concentration, which was observed when the initial glucose concentration increased, was previously explained by the need for glycerol as an osmotic regulator due to the high osmotic stress at high glucose concentrations.^34^ Under anaerobic conditions when the respiratory system is not functioning, the production of biomass and organic acids is accompanied by the net formation of NADH, which must be reoxidized to NAD^+^ by the formation of glycerol to reduce the imbalance in the NAD^+^/NADH ratio. The formation of 1 mol of glycerol during alcoholic fermentation reoxidizes 1 mol of NADH. Under osmotic stress conditions, the formed glycerol accumulates inside the cell where it plays a role in antilysis to protect the cell.^37^ A high glucose concentration may lead to high osmotic stress and more organic acid production, which increases the need for more glycerol.

**Figure 4:**
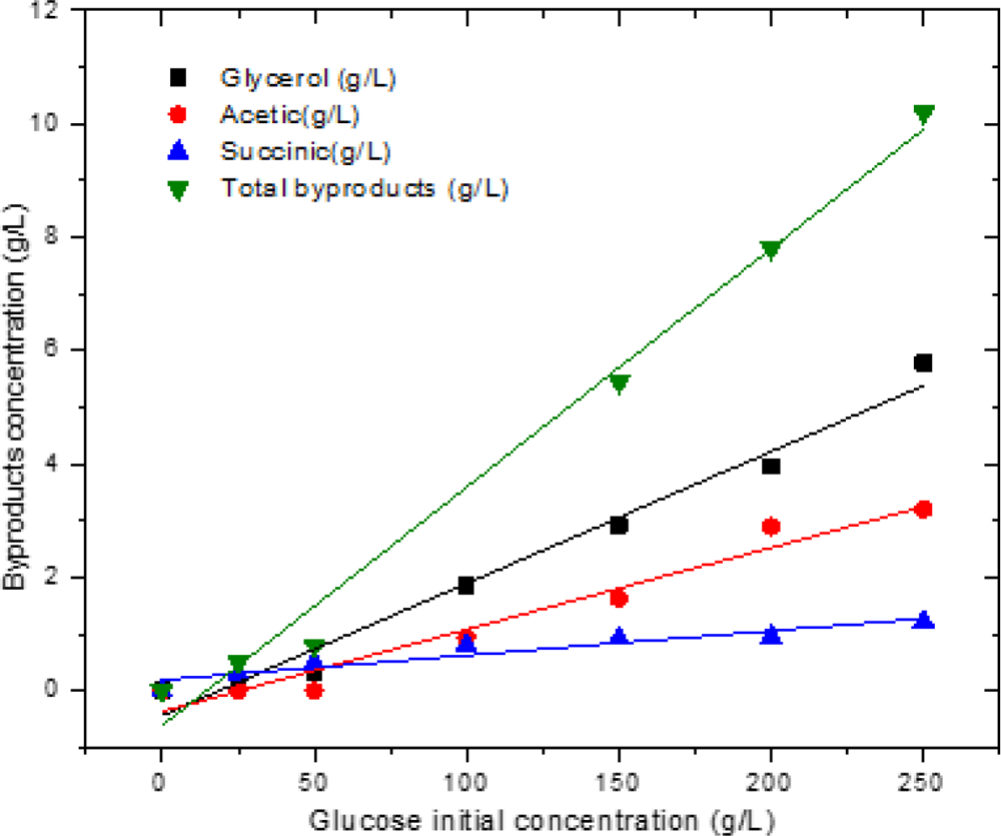
Effect of initial glucose concentration on byproduct formation during alcoholic fermentation.

Acetic acid showed a similar response. The quantity of acetic acid produced at 100 g/L glucose concentration doubled and reached 2.89 g/L when the glucose concentration was doubled. Acetate is formed as a byproduct of yeast metabolism where it is an intermediate of the acetyl-CoA synthesis pathway from acetaldehyde, which is considered to be the main source of acetate.^38^ Even though succinic acid formation increased with the increase in initial glucose concentration, it showed less dependency on glucose concentration compared to the other byproducts. Succinate is an intermediate of at least four metabolic pathways within the yeast; however, the tricarboxylic acid cycle (Krebs cycle) is the main pathway of succinic acid production during alcoholic fermentation.^39^ As depicted in Figure 4, there was a linear relationship between byproduct formation and initial sugar concentration. The yields of total byproducts, glycerol, acetic acid, and succinic acid per gram of glucose were 0.0442, 0.023, 0.0155, and 0.0054, respectively.

### Effect of Byproducts on Alcoholic Fermentation

**Figure 5:**
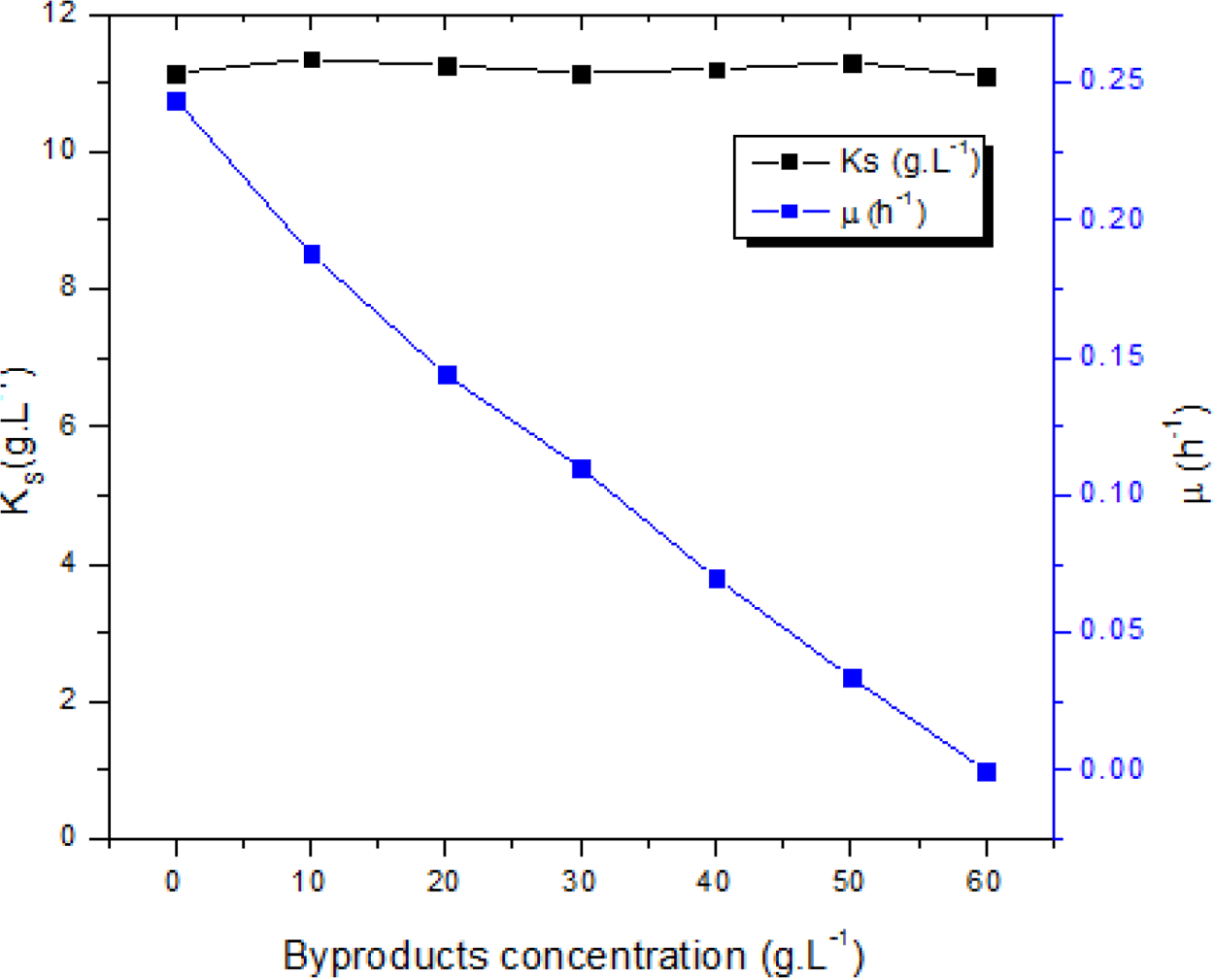
Effect of byproduct concentration on half-saturation constant and specific growth coefficient.

The biological effect of inhibitors on microorganisms can be synergistic or antagonistic by the presence of other inhibitors, which means that the effect may be significantly enhanced or reduced more than expected from individual inhibitor.^40^ This confirms the need to take the associated effect of substances into account during the investigation of their inhibitory effects rather than considering the specific inhibition of each substance. In this regard, a mixture of byproducts was prepared on the basis of the first experiment (56% glycerol, 36% acetic acid, and 8% succinic acid) at different concentrations (0–60 g/L) and added to the fermentation medium (*S*_0_ = 25 g/L) at the beginning of fermentation to investigate the effect of the byproduct mixture concentration on the fermentation process in terms of specific growth coefficient (μ_max_) and the half-saturation constant (*K*_s_). The initial concentration of glucose in the medium was 25 g/L to avoid substrate inhibition and ethanol inhibition, which may affect the reliability of the experimental data of the byproduct inhibition. Figure 5 represents the effect of byproduct mixture concentration on both specific growth coefficient (μ_max_) and half-saturation constant (*K*_s_).

The results showed that byproduct concentration has no significant effect on the half-velocity constant (*K*_s_). A slight independent change was noted in *K*_s_ values, which ranged between 11.4 to 11.7 g/L. On the other hand, the byproduct concentration showed a significant effect on the maximum specific growth coefficient. The effect of byproducts on specific growth coefficient was considerable in the range of 0–60 g/L, where it sharply declined from 0.244 to reach μ_max_. = 0 (total inhibition of alcoholic fermentation) at *Z* = 60 g/L. At this point, a new coefficient was identified and called “*Z*_m_”, which represents the value of byproduct concentration where the fermentation stops and total inhibition was achieved. In the present study, the value of the maximum inhibition byproduct concentration (*Z*_m_) was 60 g/L. This remarkable inhibitory effect of byproducts, which was observed during the batch fermentation, is expected to be more significant during long-term continuous fermentation or fed-batch fermentation due to the accumulation of byproducts. Although several studies discussed byproduct formation mechanisms especially glycerol and organic acids, the inhibitory effect of these byproducts on alcoholic fermentation did not receive enough attention. Converti et al.^24^ reported that high glycerol levels lead to an anomalous excessive increase in viscosity, which could affect the product release by the cells. According to this hypothesis, the diffusion of ethanol through the cell wall could become the limiting step and the maximum specific productivity would sharply fall with increasing viscosity. Two mechanisms have been proposed to explain the inhibitory effect of organic acids: uncoupling and intracellular anion accumulation.^41^ According to the uncoupling theory, the drop in intracellular pH resulting from the inflow of organic acids is neutralized by the action of the plasma membrane ATPase, which pumps protons out of the cell at the expense of ATP hydrolysis.^42,43^ At high acid concentrations, the proton pumping capacity of the cell is decreased, resulting in the depletion of the ATP content, dissipation of the proton motive force, and acidification of the cytoplasm according to this theory. According to the anion accumulation theory, the anionic form of the acid is captured in the cell and undissociated acid will diffuse into the cell until equilibrium is reached.^44^ Pampulha and Loureiro-Dias^45^ have investigated the activity of glycolytic enzymes in the presence of acetic acid, showing that enolase was the most sensitive enzyme and that the inhibition was due to both internal acidification and direct interference with the acid. These studies showed a good contribution to clarify the nature of inhibition of glycerol and organic acid during alcoholic fermentation. Nevertheless, further efforts should be devoted to the investigation of the reaction mechanism of inhibition of these byproducts on yeast growth, which may help to prevent or reduce their inhibitory effect.

### Model Development and Modification of the Monod Model

In modern approaches to fermentation control, a reasonably accurate mathematical model of the reaction and reactor environment is required. Using process models, we can progress beyond environmental control of bioreactors into the realm of direct biological control. Development of fermentation models is aided by information from measurements taken during process operation.^46^ It is known that the complexity in a mathematical model may increase with the inclusion of environmental conditions such as multisubstrate consumption, pH change during fermentation, variable temperature, rheological changes in culture media, multiphasic environmental variability, and nonideality of mixing and stirring.^47^ In this study, the fermentation process kinetics was described with a modified Monod-type cell growth model that accounts for byproduct inhibition. Starting from the Monod equation for cell growth (eq 1), three inhibition functions were considered in modeling byproduct inhibition: linear, parabolic, and exponential. The same approach has been previously used by Luong^17^ to develop a model for the ethanol inhibitory effect during alcoholic fermentation.

### Kinetics of the Effect of Byproducts on Alcoholic Fermentation

Based on the results of studying the effect of byproducts on alcoholic fermentation, the modification of the Monod model will consider only the expression of the maximum specific growth coefficient (μ_max_) as the byproduct concentration did not show any effect on the half-velocity constant *K*_s_, as mentioned earlier.

Considering the new equation of the specific growth under byproduct inhibition, the new modified Monod model will be written as follows:
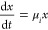
7

**Figure 6:**
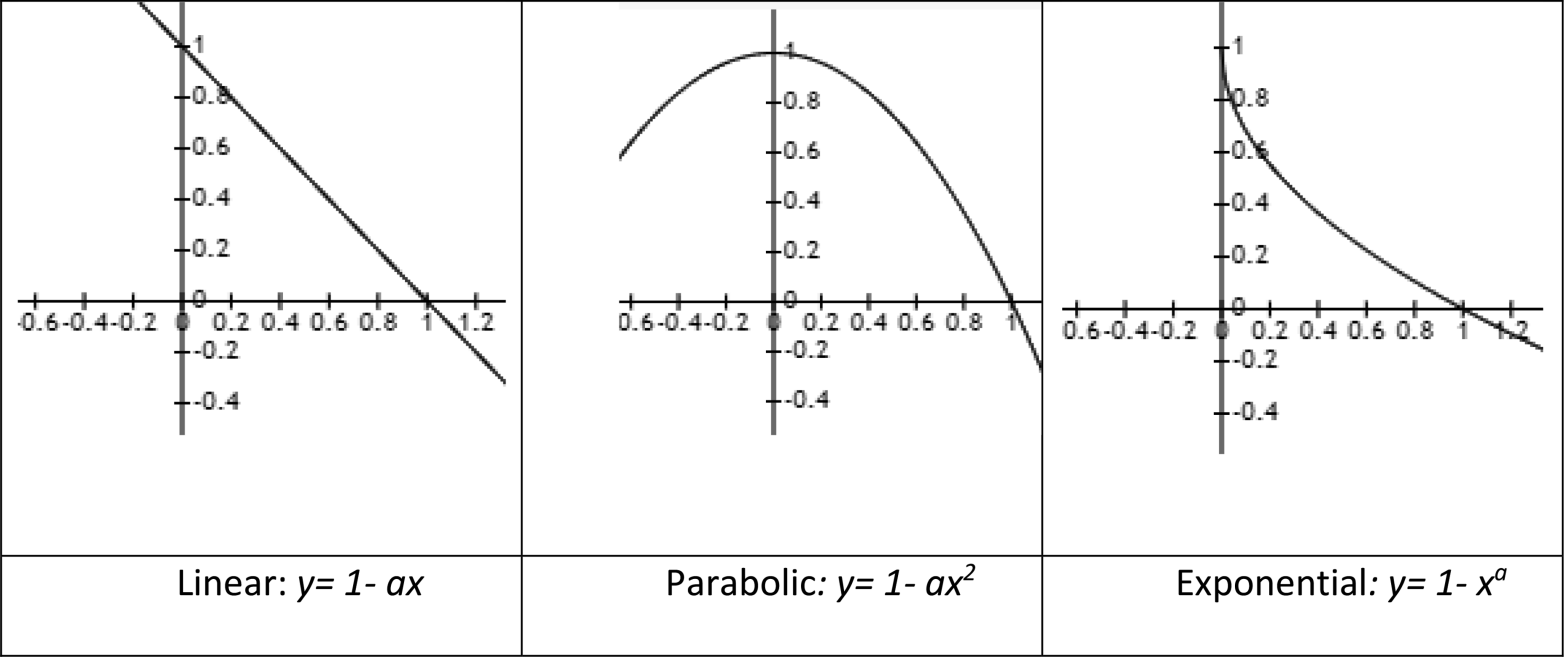
Plots of the proposed functions for the byproduct inhibitory effect.

To develop the new modified model, it is required to define the inhibition function described in eq 2 and to calculate the values of yield coefficients. Defining μ_max0_ as the specific growth rate without byproduct inhibition (*Z* = 0), *Z*_max_ as the concentration at maximum byproduct inhibition (μ_max_ = 0), and *K*_z_ as the byproduct inhibition coefficient, it can be assumed that  when  and  when  →1. Thus, the limitations of the inhibition functions can be defined as follows:
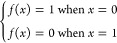
Through the screening of the library functions of the Origin Pro 8.5 software, the three equations represented in Figure 6, which verify the limitations, have been selected to be fitted against the experimental data for the identification of the byproduct inhibition function.

By substituting the variable (*x*) with  and the constant (*a*) with the byproduct inhibition coefficient (*K*_z_), the three mathematical expressions can be written as follows:
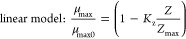

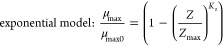

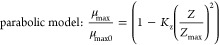


**Figure 7:**
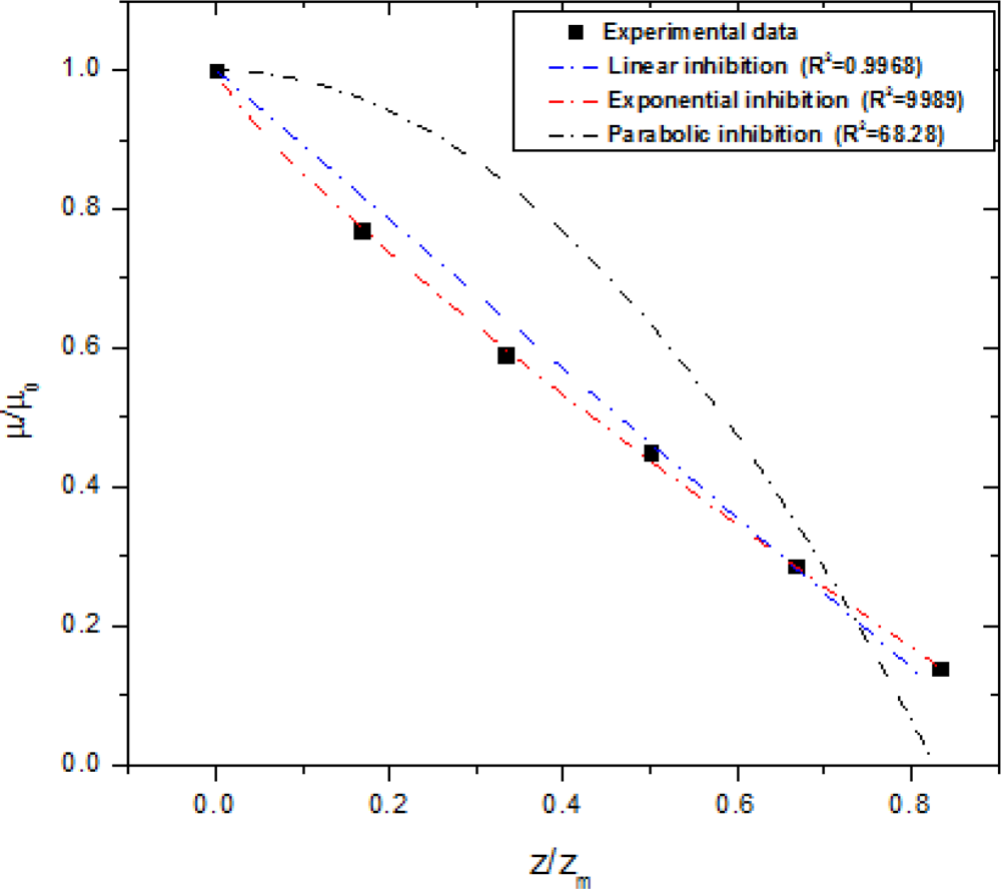
Models fitting to the byproduct inhibitory effect during alcoholic fermentation.

The experimental data were fitted using the OriginPro 8.5 software to the three proposed models, as shown in Figure 7. It was found that the exponential model showed good agreement with the experimental data with the highest R-square (*R*^2^ = 0.9989). On the other hand, the linear model also showed a good fitting to the experimental data (*R*^2^ = 0.9968) compared to the exponential model. In contrast with the two other models, the parabolic model seems to be not suitable to present the experimental data with a lower *R*-squared (*R*^2^ = 0.6828).

Based on the fitting results, the exponential model will be considered as the suggested model to describe the inhibitory effect of byproducts during fermentation, Equation 3 can be written as follows:
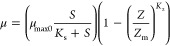
8*Z*_m_ is the maximum byproduct concentration where the fermentation stops totally.

*K*_z_ is the byproduct inhibition coefficient, which is mainly dependent on yeast strain and operating conditions.

In the present study, the fitting of the experimental data showed that *Z*_m_ = 60 g/L and *K*_z_ = 0.83.

### Calculation of Yield Coefficients

The cell mass yield coefficient *Y_x/s_*, the product yield coefficient *Y_p/s_*, and the product yield coefficient can be calculated during the growth phase based upon the parallel conversion stoichiometry equations 4–6.

The values of *Y_x/s_*, *Y*_p/s_, and *Y_z/s_* were calculated in the range between 25 and 250 g/L initial substrate concentration (*S*_0_). No significant change in both yield coefficients was observed in the selected range and the average values of *Y_x/s_*, *Y*_*p/s*,_*Y_z/s_* were found to be 0.28, 0.42, and 0.0442, respectively, as shown in Table 1.

**Table 1:** Calculation of the Average Values of the Yield Coefficients

*S*_0_	*Y_x/s_*	*Y_p/s_*	*Y_z/s_*
25	0.27	0.39	0.0427
50	0.28	0.43	0.0432
100	0.27	0.41	0.0441
150	0.29	0.42	0.0445
200	0.30	0.44	0.0453
250	0.27	0.43	0.0456
average	0.28	0.42	0.0442

By substituting the calculated values of μ_max_, *K*_s,_ and *K*_z_ in eq 8 and the calculated values of *Y_x/s_*, *Y_p/s_*, and *Y_z/s_* in eqs 4–6, respectively, the new modified model considering the byproduct inhibitory effect will be as follows:
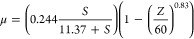
9
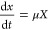
10
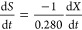
11
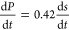
12
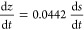
13

### Simulation of the Proposed Model and Comparison with the Conventional Monod Model

**Figure 8:**
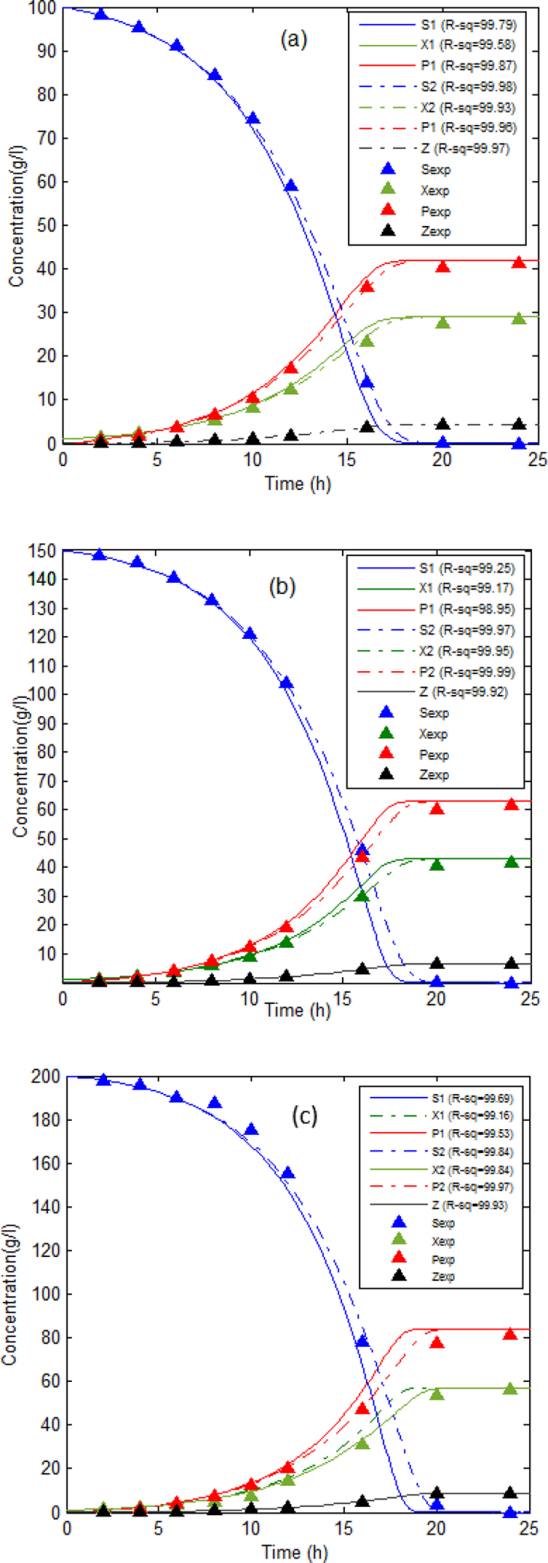
Comparison between the simulation data and the experimental data at different initial substrate concentrations: (a) 100 g/L, (b) 150 g/L, and (c) 200 g/L. X_1_, S_1_, and P_1_ are the simulation data of mass cells, substrate, and product concentrations, respectively, using classical the Monod model. X_2_, S_2_, P_2_, and Z are the simulation data of mass cells, substrate, product, and byproduct concentrations, respectively, using the new modified model. X_exp_, S_exp_, P_exp_, and Z_exp_ are the experimental data of mass cells, substrate, product, and byproduct concentrations, respectively.

Simulation of the new modified model and the Monod conventional model, which were developed using experimental data, was conducted using the MATLAB R2014a software. Simulation data of both models were compared to the experimental data for a batch fermentation at different initial substrate concentrations (*S*_0_ = 100 g/L, *S*_0_ = 150 g/L, and *S*_0_ = 200 g/L), as presented in Figure 8.

In all the presented figures, both predicted data of the modified model and Monod model were in good agreement with the experimental data in the first stage of the fermentation. Progressively, a remarkable decrease in glucose consumption, ethanol production, and biomass production was observed in the modified model data compared to Monod data; this difference between both models increased by the time driven by the accumulation of the byproducts in the fermentation broth, which increases its inhibitory effects.

On the other hand, the simulation data of the Monod model are in good agreement with the simulation data of the proposed model at low concentrations. However, this difference became more significant at high concentrations as the gap between the two models is increasing with the increase in initial substrate concentration. This is expected as the formation of byproducts mainly depends on the consumption of the substrate rate (which leads to a higher inhibitory effect). Consequently, a delay in the predicted fermentation time was observed in the modified model compared to the fermentation time in the conventional Monod model. This delay increased with an increase in the initial substrate concentration where it was estimated to be around 0.5 h for *S*_0_ = 100 g/L, 1 h for *S*_0_ = 150 g/L, and 2 h for *S*_0_ = 200 g/L. The validation of both models against the experimental data at different initial glucose concentrations showed the ability of the developed model to represent the inhibitory effect of the byproducts, which has been reflected to have higher accuracy compared to the classical Monod model.

The average absolute deviation values for the modified model were 67.14, 18.59, and 26.60 for the substrate concentration, biomass concentration, ethanol concentration, respectively; meanwhile, the conventional Monod model had average absolute deviation values of 70.07, 17.28, and 27.69 for the substrate concentration, biomass concentration, ethanol concentration, respectively.

For more reliability, the accuracy of the modified model and Monod model was also evaluated in terms of mean square error (MSE) and root-mean-square error (RMSE) for predicting the concentrations of biomass, substrate, and products, as shown in Table 2. The results showed lower (MSE) and RMSE of the modified model compared to the Monod model. These findings confirmed the results reported in the validation of the model using the coefficients of determination (*R*^2^), which indicated high accuracy and predictive power of the proposed modified model compared to the conventional Monod model. The results of the validation revealed that incorporating the inhibitory effect of byproducts has significantly optimized the accuracy and the performance of the model in predicting the fermentation data.

**Table 2:** Summary of Statistical Analysis of the Models

statistical test	variable	modified model 2	monod model
MSE	substrate	15.83	40.73
	biomass	2.19	8.75
	product	3.88	16.16
	byproducts	0.02	
RMSE	substrate	3.97	6.38
	biomass	1.48	2.95
	product	1.97	4.02
	byproducts	0.15	

Overall, it is evident that the inhibitory effect of byproducts increases with the time and the consumption of the substrate. As the continuous fermentation characterized by the long-term fermentation process results in higher accumulated byproduct concentration than the batch fermentation, it is predicted that the difference between the modified model data and Monod model data will be more significant.

## Conclusions

In the present study, glycerol and weak organic acids including acetic and succinic acid were the major byproducts during the fermentation process; however, no lactic acid was detected in the fermentation broth, which confirms that lactic acid is a product of contaminated fermentation only. During the batch fermentation, the formation of these byproducts is mainly related to the initial substrate concentration. Despite the fact that byproduct concentration does not reach a point where it may stop totally the batch fermentation, the accumulation of these byproducts may significantly inhibit the yeast growth and slow down the fermentation, which may decrease the productivity. Indeed, the new developed model, which takes the inhibitory effect of these byproducts into consideration during the modeling of alcoholic fermentation, will efficiently help in controlling and optimizing the fermentation process. However, more efforts should be devoted toward the investigation of the mechanism of this inhibitory effect to develop new strategies to limit it.
